# Il-6 is non-essential to murine CD19 CAR efficacy, but can mediate acute GVHD following allogeneic BMT with CAR T cell infusion

**DOI:** 10.1186/2051-1426-3-S2-P226

**Published:** 2015-11-04

**Authors:** Elad Jacoby, Haiying Qin, James Kochenderfer, Yinmeng Yang, Chris Chien, Terry Fry

**Affiliations:** 1 Pediatric Oncology Branch, National Cancer Institute, Bethesda, MD, USA; 2 NCI, NIH, Bethesda, MD, USA; 3 National Cancer Institute, Bethesda, MD, USA; 4 Pediatric Oncology Branch, CCR, NCI, NIH, Bethesda, MD, USA

## 

Chimeric-antigen-receptor (CAR) T cells targeting CD19 show dramatic remissions in refractory or relapsed acute lymphoblastic leukemia. Interleukin-6 (IL-6) has been associated with severe cytokine release syndrome (CRS) following CAR T cell treatment, leading to significant toxicity and death. CRS could be treated with an anti-IL6-receptor antibody, with reversal of most of the inflammatory response within hours to days. In addition, the significance of IL-6 in prevention and treatment of graft-versus-host disease (GVHD) following allogeneic bone marrow transplant (allo-BMT) has been shown in pre-clinical and clinical settings.

Here, we used an immunocompetent murine model of ALL to study CD19 CAR biology. In a syngeneic/autologous model, we found no significant increase in IL-6 in leukemia bearing mice following CD19 CAR treatment. No significant alteration in efficacy was seen when administering CD19 CAR from wild-type mice or IL6-/-mice to wild-type or IL6-/-recipients, with all achieving long term remissions compared to controls.

To elucidate IL-6 in this system, we used a minor-mismatch allo-BMT mouse model (B6àC3h.sw), previously shown to have increased IL-6 levels when T cell replete BMT given. Using a T cell depleted allo-BMT platform, we saw significant increase in IL-6 in leukemia bearing mice treated with CD19 CAR T cells compared to leukemia-bearing controls treated with mock-T cells, and non-leukemia bearing allogeneic CAR recipients. Peak of IL-6 levels was seen 5 days following CAR-T cell infusion, either when given in proximity to the BMT or in later time points. The rise in IL-6 correlated with an acute systemic inflammatory process resembling acute GVHD, that caused early lethality despite being a minor-mismatch model, and at relatively low T cell doses. This acute GVHD could not be reversed when T cells were given early (day 0-2 post BMT), using either IL6-receptor blockade or IL6-/-bone marrow on day 0. However, when leukemia and CD19 CAR administered late (day +12-+17) following allo-BMT, wild-type marrow recipients still died of acute GVHD while IL6-/-recipients were rescued.

Altogether, we show that IL-6 is not essential for CD19 CAR efficacy in this murine model, but can drive significant toxicity following an allogeneic BMT with CAR.

